# Mind, body, health: qualitative insights into a holistic health concept for preschoolers

**DOI:** 10.3389/fmed.2026.1911127

**Published:** 2026-09-16

**Authors:** Marinela Gerganova, Angela Tekath, Georg Seifert, Wiebke Stritter, Sarah B. Blakeslee

**Affiliations:** 1 Charité Competence Center for Traditional and Integrative Medicine (CCCTIM), Charité – University Medical Center Berlin, Corporate Member of Freie Universität Berlin and Humboldt Universität zu Berlin, Berlin, Germany; 2 Department of Pediatric Oncology and Hematology, Charité – University Medical Center Berlin, Corporate Member of Freie Universität Berlin and Humboldt Universität zu Berlin, Berlin, Germany

**Keywords:** health promotion, holistic program, kindergarten, Kneipp, pre school, resilience, salutogenesis, water

## Abstract

**Introduction:**

The German holistic health Kneipp concept (KC) consists of five elements: water applications, physical activity, healthy nutrition, herbs, and mind–body practices. Well-known in German-speaking countries, the program’s effects in early childhood education are little known. This analysis examined the qualitative portion of the larger mixed-method *Kita Kneipp Study* exploring the potential and challenges of implementing Kneipp in kindergartens to promote salutogenesis and prevent illness in children through teachers’ and children’s experiences.

**Methods:**

Semi-structured Expert interviews with teachers and field notes from focused ethnographies of Kneipp activities in kindergartens were used to triangulate data. Data were then thematically analyzed.

**Results:**

Teachers observed improved resilience to cold and calming effects of the interventions. Children learned to support their own and others’ wellbeing by engaging in activities. Developing bodily awareness, Kneipp helped with self-regulation, promoted curiosity, self-confidence, and the ability to set boundaries. Acceptance by teachers, children, and parents indicated potential for long-term use. Reported challenges included initial skepticism among teachers and children. Structural issues like staff shortages, limited equipment, and low initial teachers’ involvement were also mentioned. Parental misunderstandings, especially in low socio-economic areas, highlighted the importance of proactive communication and family involvement.

**Discussion:**

The KC can be integrated into kindergartens, supporting physical and mental health while fostering mindfulness, social competence, and resilience. The study shows high acceptance and meaningful developmental benefits, with potential for sustainable implementation in early educational settings. These aspects may positively influence health promotion from an early age.

## Graphical abstract

**Figure:**
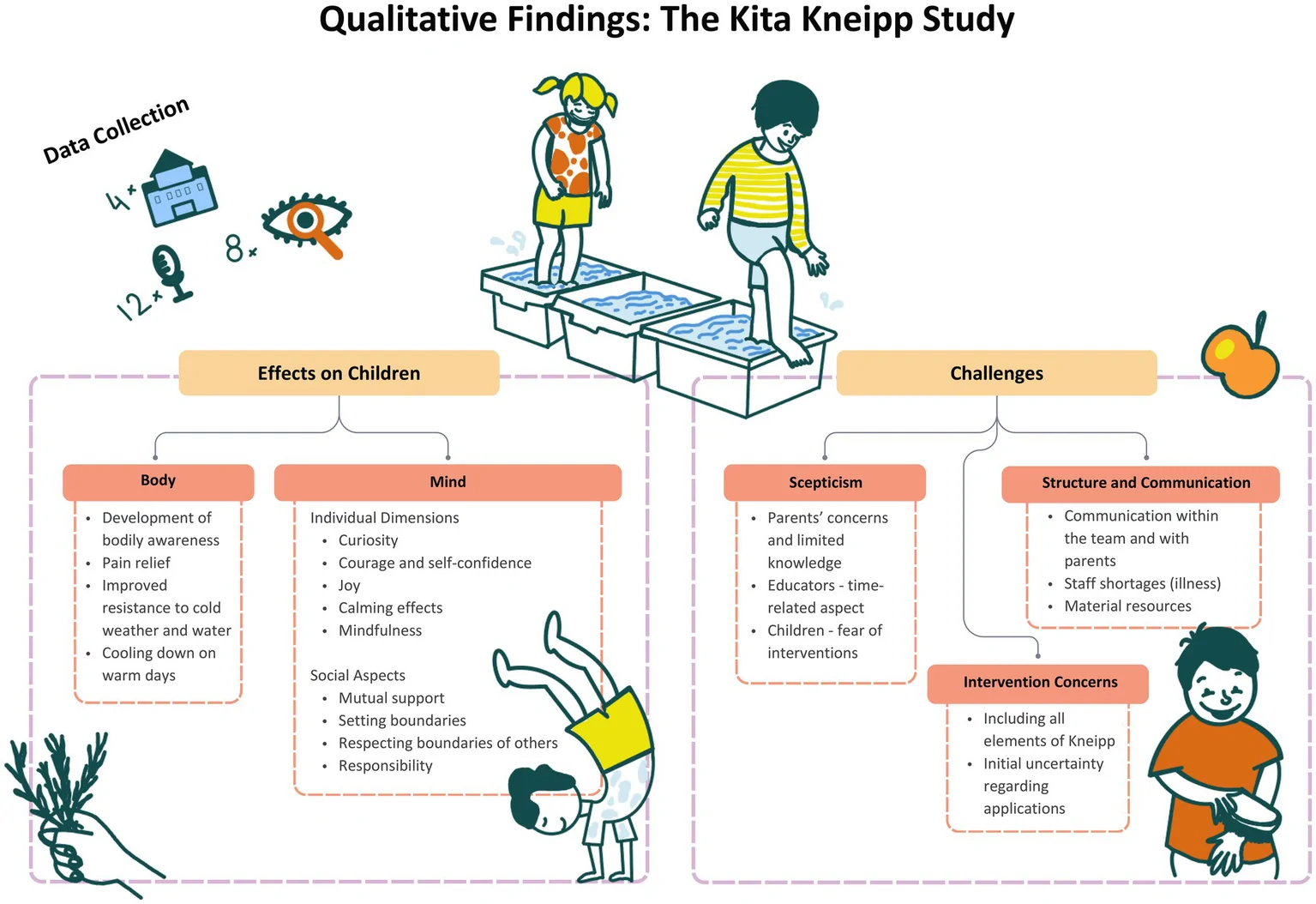

## 1 Introduction

*“Health is created and lived by people within the settings of their everyday life; where they learn, work, play and love. Health is created by caring for oneself and others, by being able to take decisions and have control over one’s life circumstances…”* (1).

Early childhood is a critical period for establishing lifelong habits, making it a key phase for promoting health and wellbeing (2, 3). Children, particularly in this developmental stage, are considered one of the most vulnerable population groups. This vulnerability stems from a combination of biological, developmental, and social factors, including their developing immune systems, susceptibility to infections, and dependence on adults for fulfilling basic needs (4). Additionally, socioeconomic disparities can further influence children’s overall wellbeing and development (2, 5). Therefore, finding solutions to reduce inequities is a fundamental factor to achieving the Sustainable Development Goals (SDGs) of the United Nations, aiming to address global challenges, including, for instance, health, education and poverty, all vital factors for addressing primary health (6, 7).

Research demonstrates that early experiences fundamentally alter the brain structure and function during critical developmental periods where the brain is most plastic and responsive to environmental input. These early life events have a powerful influence on both the pattern of brain architecture and behavioral development (8). In line with these findings, health prevention from an early age is increasingly recognized as essential, as multiple factors influence children’s long-term health and wellbeing (9, 10).

Beyond the family environment (11), institutions such as kindergartens play a pivotal role in shaping children’s physical, mental, and social development (12, 13). In these settings, children not only acquire knowledge but also form the foundations of lasting health behaviors. As such, it is vital that early childhood institutions provide an environment that fosters the development of these essential habits for lifelong health (14).

The importance of this is listed as a key point in German law on prevention (15). This also aligns with Antonovsky’s Salutogenesis Model with its focus of developing a Sense of Coherence (SOC), consisting of comprehensibility, manageability, and meaningfulness which describe an individual’s capacity to perceive life as structured, manageable, and worthy of engagement (16, 17). The development of SOC is supported by factors such as social support, stable environments, participation opportunities and access to resources. Early childhood education and care institutions provide key settings for strengthening resilience and fostering salutogenic strategies from an early age. Despite this, holistic approaches to health are uncommon in these settings, seldom addressing mental and social health along with bolstered salutogenesis and resilience (18).

The Kneipp Concept (KC), is a well-established holistic health approach stemming from Kneipp therapy that was developed by the German priest Sebastian Kneipp in the 19th century. It integrates five core elements: hydrotherapy with focus on cold water applications, physical exercise, wholesome, plant-based nutrition emphasizing regional and seasonal foods, the use of medicinal herbs, and mind–body practices (19). Kneipp has been found to alleviate some symptoms associated with conditions such as chronic obstructive pulmonary diseases, cardiovascular diseases, sleep disorders, and menopausal symptoms in adult populations (20). The goal of using Kneipp with children is to foster self-awareness of both physical and psychological needs and behaviors that support long-term health and wellbeing. In children, water applications include cold water exposure to the surface of the skin through arm and leg pourings or immersions, creating a water shield to cover the limbs to stimulate vascular and thermal function. Further contrasting cold and warm baths are used. The physical activity element encourages both structured exercise and the integration of functional movement into everyday life to support overall physical fitness and health that promotes feeling within the own body. Nutrition represents another central element of the KC, promoting a balanced, plant-based diet based on regional and seasonal products. The use of herbs as an element combines a supportive health measure against illness, for instance teas for coughs or sore throats, and as an aid to wellness, such as with lavender oil to promote relaxation. The umbrella element mind–body approaches focus on maintaining a healthy balance between periods of activity and recovery. Recommended practices include stress management strategies such as relaxation techniques, meditation, and breathing exercises, as well as ensuring adequate sleep, and sufficient time for rest and recovery (21). For better understanding on how these elements were put into practice with children, we created a short film about the concept in the kindergarten setting which can be found under the following link: https://player.vimeo.com/video/1193903554 (22).

The KC offers a comprehensive approach to health that aims to address both the physical and mental needs of children, potentially supporting their development of resilience and health awareness from an early age and is being practiced in more than 500 kindergarten facilities in Germany (23). However, the need for teachers’ training, their motivation to carry out added interventions within an already established daily kindergarten routine and needed resource allocations for conducting new practices might appear challenging in the implementational process of such concepts, as was also found in another study which explored the integration of a healthy nutrition program to kindergartens for obesity prevention. There, challenges such as skepticism of teachers or sensitive topics with parents occurred showing the relevance of addressing these when introducing new concepts (24). Therefore, it is necessary to explore how the KC can be successfully and sustainably implemented in kindergartens, and what its effects may be on children’s overall health and wellbeing to promote positive lifelong habits.

The Kita Kneipp Study is a mixed-method study, analyzing quantitative and qualitative data related to the Kneipp concept and its’ implementation in kindergartens. The overall study assessed whether Kneipp interventions reduced sick days and illness symptoms in children compared to a control group and these findings are reported separately (25). The present paper focuses on specific insights gained through qualitative data consisting of interviews with teachers and directors and focused ethnographies, addressing following research questions:

What *potential* and *possible challenges* are there to implementing a holistic Kneipp concept in kindergartens for *illness prevention* and *salutogenesis promotion* in children?

- How does the KC affect the physical, emotional, and social wellbeing of children?
- To what extent can the KC be aligned with an existing educational framework in kindergarten facility in the context of illness prevention and salutogenesis?
- Which factors may contribute to the successful long-term implementation of the Kneipp approach as a health promotion program in childcare facilities?

All research questions were informed by both qualitative data sources. Interviews with teachers and directors provided participants’ experiences and perceptions, while the focused ethnographies contextualized and enriched these accounts through direct observation of everyday practice. Together, these complementary data sources enabled a comprehensive understanding of the concept implementation, its perceived health-promoting potential, and the factors influencing its sustainable integration into kindergarten settings.

## 2 Materials and methods

### 2.1 Study design and setting

This present paper reports on the qualitative portion of the Kita Kneipp Study, which has a confirmatory, mixed-method, two-armed, waitlist, cluster-randomized controlled trial design (registered with the German Clinical Trial Register (DRKS-ID: DRKS00029275, on November 10, 2022, approved by the Ethics Committee of Charité - Universitätsmedizin Berlin on September 13, 2022, Application number: EA2/122/22). It follows the standards of the Helsinki convention of good clinical practices. Details of the overall study design are described in the published study protocol (26). The qualitative data, the purpose of which was to examine how the KC intervention was implemented, accepted and experienced within the kindergartens, consisted of expert interviews and focused ethnographies in the intervention group facilities after commencement of Kneipp activities between February 2023 and September 2024.

The recruiting process, consent materials, data storage, inclusion, and exclusion criteria were conducted as described in the study protocol (26). All participants, including parents/guardians, directors and teachers, gave their written consent after being briefed about the study’s purposes. The results are reported according to the qualitative research COREQ-Checklist (see Supplementary material – COREQ Checklist) (27).

### 2.2 Sampling, data collection, and analysis

The kindergarten intervention group included four different socio-demographic regions in Berlin: Western Central Berlin (WCB), Eastern District Berlin (EDB), Eastern Central Berlin (ECB), and Surrounding Berlin Area (SBA). As part of the overall mixed-method design, after quantitative data for the baseline was collected, a 4-day-teacher training in Kneipp for children was organized in the intervention facilities and attended by teachers and directors [training topics details as published (26)]. Following this, interviews were conducted after starting Kneipp activities with all directors and strategically selected teachers based on a maximum variation sampling of experience, age and gender. Two focused ethnographies were planned in each kindergarten of the intervention group in opposing seasons after establishing Kneipp practices within the kindergarten routines, to examine the implementation of the interventions, allowing for seasonal comparisons of activities. The qualitative data collection was conducted by two study team members (MG – a female medical doctoral student planning her career in the pediatric field, and with interest in salutogenesis and health promotion and SB – a female qualitative expert, with a focus of research on health promotion and illness prevention in pediatrics). Kindergartens were asked to focus on water applications, while integrating all Kneipp elements into their individual concepts.

Qualitative findings were thematically analyzed (MG) according to Kuckartz to capture diverse perspectives and structure the findings meaningfully using the MAXQDA 2024 (VERBI Software, Release 24.2.0) (28). First deductive categories were developed from the interview guideline. Main participant topics were summarized and analyzed for the development of further inductive categories. Drafted memos further aided reflection and data interpretation. Findings were discussed and supervised (SB and WS – qualitative experts) in regular team meetings and additionally in a qualitative research workgroup throughout analysis, challenging assumptions, and enhancing interpretive accuracy.

#### 2.2.1 Interviews

Teachers in the German kindergarten setting spend between 6 to 9 h with the children having a good overview of the children’s pedagogical wellbeing (29). Additionally, directors have a comprehensive overview of programmatic administration and what may work within a kindergarten setting. Therefore, directors and teachers were recruited in the intervention kindergarten facilities by the study team either face-to-face during the focused ethnographies, or via telephone. Semi-structured interviews were conducted in person in the kindergartens, in German and were audio recorded (SB and MG). Recordings were auto-transcribed with the web-based software f4x (audiotranskription.de) then corrected and pseudonymized (MG). No further input was given by interviewees. Post-interview field notes were written directly after conducting the interviews by summarizing the main topics mentioned and documenting contextual information (e.g., time, setting, and interview atmosphere). These field notes were used to provide information during the analysis, to support reflexivity throughout the process, and for the improvement of subsequent interviews. Variables based on gender, age, and work experience were included in the analysis, comparing cases for a better understanding of different experiences with the concept and points of view.

#### 2.2.2 Focused ethnographies

This approach aimed not only to explore the effects of the KC, but also adaptability and practical feasibility across seasons. This method was particularly suitable for research involving young children, as focused ethnographies enabled the observation of children’s experiences and behaviors within their natural kindergarten environment and allowed direct exchange, understanding better their experiences with the activities. Since young children may have limited ability to verbally express their perceptions, feelings, or responses to the Kneipp interventions, direct participative observation provided valuable insights into their engagement, reactions, and interactions (30, 31). Focus on the implementation process, sought to determine KC sustainability and its application to year-round health-promotion, understanding children’s experience in an age-appropriate way (32). Ethnographic field notes were recorded during each observation to document on-site impressions, contextual information, and observed interactions. These notes were used to develop the Post Ethnography Summaries (PESs), which formed the basis for the initial analysis of the ethnographic data (see Supplementary material – Example of a Field Note). After initial coding tree was established from interviews it was applied to PESs. Analysis between interviews and ethnographies used axial coding to achieve methodological triangulation by comparing findings across both data sources, allowing the validation of the inductive codes and the emergence of analytical categories answering the central research question.

## 3 Results

### 3.1 Participants’ description and observed interventions

We conducted 12 interviews (3 per facility), with each of the intervention kindergarten directors and two teachers, with a duration between 37 min and 1 h 14 min. All the interviews took place at least 15 weeks after starting with Kneipp activities in the kindergarten facilities without the presence of others. Table 1: Participants’ Characteristics presents the characteristics of the interview participants, including gender, age range, years of work experience, and their position within the kindergarten. None of the contacted teachers refused to take part in an interview. Eight focused observations (2 per facility) with a mean duration of 2.8 h were conducted during opposing seasons (spring/summer and autumn/winter) at least 13 weeks after introducing Kneipp in the facilities to examine the implementation of the interventions, allowing for seasonal comparisons of activities. Details about the conducted focused ethnographies regarding month and year, duration, and observed interventions are found in Table 2.

**Table 1:** Participants’ characteristics.*

Interview participant	Age range	Gender	Position in the facility	Work experience
WCB5	55–59	m	Director	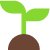
WCB2	50–54	m	Teacher	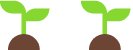
WCB4	60–64	f	Teacher	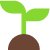
EDB7	35–39	f	Director	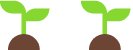
EDB1	50–54	f	Teacher	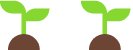
EDB10	45–49	f	Teacher	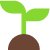
ECB12	45–49	f	Director	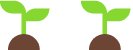
ECB11	40–44	f	Teacher	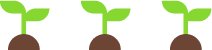
ECB9	25–29	m	Teacher	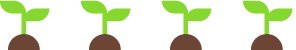
*SBA*3	35–39	f	Director	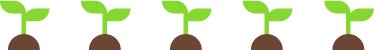
*SBA*6	35–39	f	Teacher	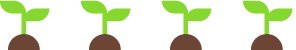
*SBA*8	30–34	f	Teacher	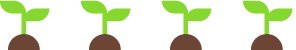

* m (male), f (female), Western Central Berlin (WCB), Eastern District Berlin (EDB), Eastern Central Berlin (ECB), Surrounding Berlin Area (SBA), Level of experience: 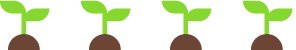 (0–5 years), 
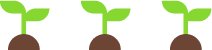
 (5–10y), 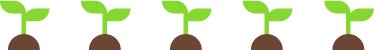 (10–15y), 
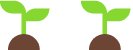
 (15–20y), 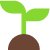 (>20y).

**Table 2:** Focused ethnographies.*

KITA	Month, year	Duration	Intervention (S)
WCB	June 2023	3 h 10 min	Water treading, Leg pouring
WCB	March 2024	2.5 h	Water treading
EDB	May 2023	3 h 15 min	Alternating feet bath, Arm bath
EDB	January 2024	3 h	Water treading, Arm bath
ECB	November 2023	2.5 h	Dry brushing massage, Aroma application
ECB	September 2024	1.5 h	Water treading
SBA	September 2023	2 h 20 min	Recognizing Herbs, Water treading
SBA	February 2024	3 h 50 min	Sauna

* Western Central Berlin (WCB), Eastern District Berlin (EDB), Eastern Central Berlin (ECB), Surrounding Berlin Area (SBA).

### 3.2 Effects of Kneipp interventions on preschool children

The Kneipp interventions were reported to have various physical and mental effects on children, contributing to health promotion and emphasizing the importance of resources and strategies that children can acquire in such settings for their future adult lives (see Figure 1: Effects of Kneipp on Children).

**Figure 1:**
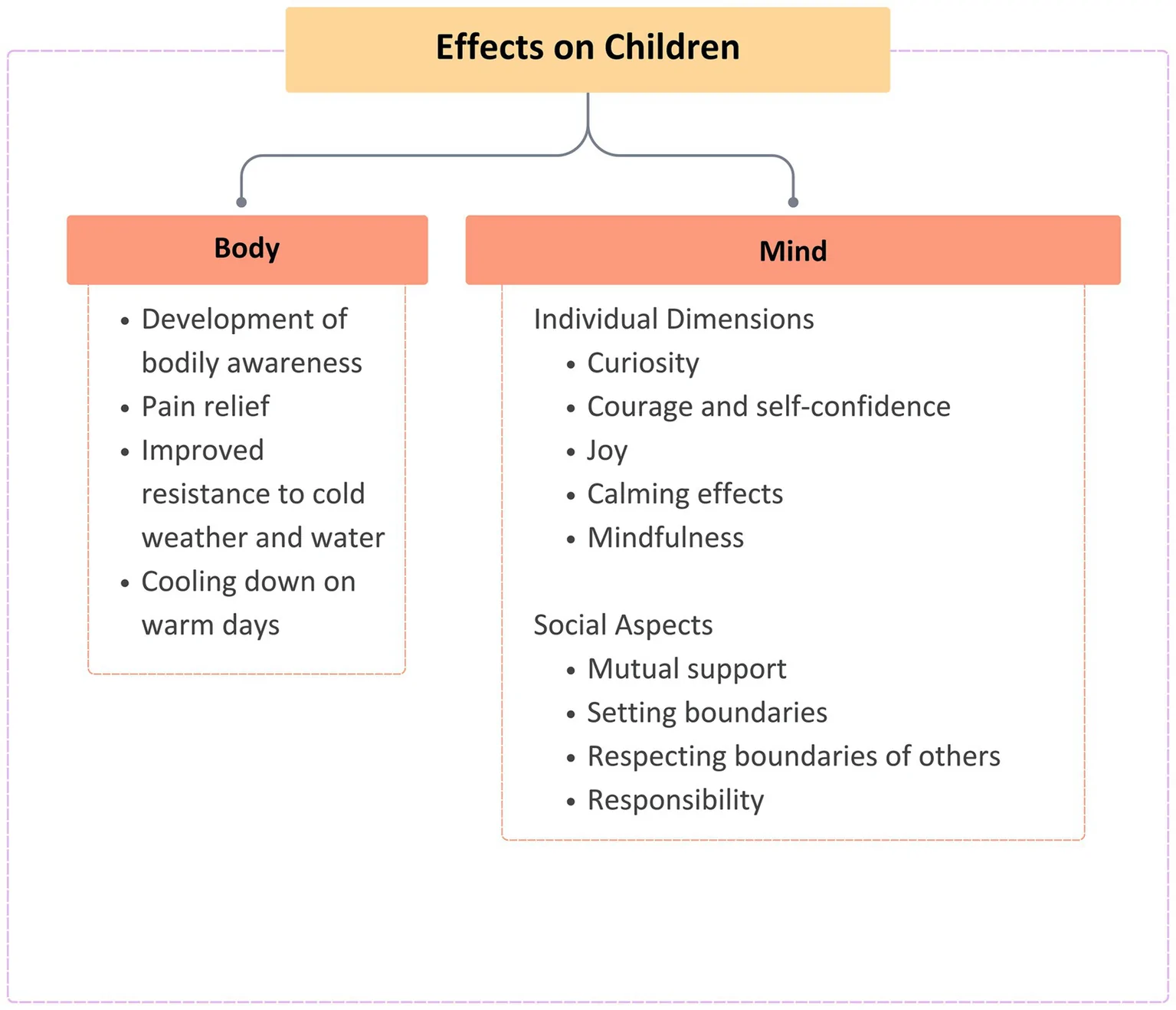
Effects of Kneipp on children – Key categories and subcategories drawn from data analysis.

#### 3.2.1 Effects on the body

In line with the salutogenic approach, teachers reported that the Kneipp interventions fostered children’s physical resilience and adaptive health resources. They observed several effects, such as increased resistance to cold weather and water, which was perceived by staff to contribute to fewer sick days during the colder seasons among children and consequently also among teachers.

*“But this training with cold water has definitely happened here. They are a bit more resilient to temperature differences, I’d say – not 100% of them, but I think they are generally more tolerant of colder water.”* – WCB2, teacher, 50–54y, 
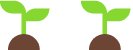
.

Physical effects of Kneipp also included children dealing with heat on particularly hot summer days with the help of cold-water Kneipp interventions, such as leg and arm baths, or facial rinses (22). Furthermore, teachers emphasized a common observation that the KC led to a greater self-awareness of children for their own body. Throughout the activities, teachers reported that during these practices, they encouraged children to reflect on their experiences by asking them what they were noticing and feeling, thereby drawing attention to bodily sensations and fostering mindfulness and self-reflection. Ethnographic observations similarly showed that children were able to verbalize their experiences of the interventions, for instance by describing the sensation of cold water, or the warmth during a sauna intervention on their skin.

#### 3.2.2 Effects on the mind

Beyond physical health benefits, mental and social effects of Kneipp were also reported in the interviews. Children were seen to recognize their own needs, taking charge of how they feel, and identifying helpful resources for challenging situations. In one kindergarten, for example, children were reported to have learned the importance of resting in order to balance emotionally overwhelming moments during play. Children’s development of early self-regulatory behaviors and preventive health skills were mentioned to be one key motivating factor for teachers to use the KC.

*“What is really important to me is that [the children] take responsibility for their own health independently, even if they are not consciously aware of it in that form. But when they notice: ‘I feel good - afterwards I feel more content, happy, joyful, relaxed, warm’, that is something where children realize: ‘I can actively take part in and influence how I feel.’ And that is exactly what we want, and that is what it [Kneipp] is for, and that is when I am thinking—it is simply great that we are able to do this.”* – WCB5, director, 55–59y, 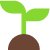

Teachers also observed that children demonstrated increased courage, self-confidence, and curiosity through their engagement in new activities. They described, for instance, how children initially approached interventions with cold water and how their responses changed over time. For example, some preschoolers were at the beginning hesitant to participate. With time, however, children were seen to have become more confident, and more curious about trying the Kneipp activities. In the ethnographic observations this was also noted.

These developments primarily reflect the manageability component of Antonovsky’s salutogenic model, with newly experienced ways to cope with new and challenging situations. This included setting personal boundaries and respecting those of their peers.

*“Of course, many factors play a role here—like safety and so on. But simply having the option to make a choice for yourself. To be able to decide: ‘Does this feel good to me or not?’ That alone is incredibly valuable, I think”* - EDB10, teacher, 45–49y, 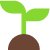

*“One child was lying there and said: ‘I can’t brush my belly; it tickles me too much - I can’t sleep afterwards.’ So, fine—then he just brushed his arm instead. That worked better for him. But even that is an important realization for the child: that brushing his belly with the brush does not feel good for him.”* - EDB7, director, 35–39y, 
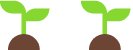

Beyond effects on one’s own wellbeing, teachers also mentioned witnessing children’s increased awareness of the wellbeing of others, particularly when practicing Kneipp interventions together in groups. Teachers highlighted that in mixed-age groups, older children often assisted younger peers when doing the interventions together, for example by counting seconds left to stay in the cold water together, or helping each other with a dry-brushing activity, used for peripheral vascular stimulation and for calming down. These were thought to strengthen social cohesion, empathy, and mutual responsibility, a point central to the concepts of salutogenesis and health promotion. The KC consequently was found to be not only a physical or self-care practice but also an opportunity to build a practice of social cohesion and provide mental resources by giving the opportunity for peers to engage in activities together, allowing them to learn to take responsibility for each other.

*“I recently observed the following situation: One child stepped into the water tub […] and bumped against the rim spilling water. Before, everyone would have stood around clapping their hands. The boy ran off, grabbed a towel, and wiped it up. He was being careful, making sure no one would slip […] And I stood there thinking: ‘Oh my God, you are four years old.’ […] He simply learned to take responsibility […] There are so many things happening with the children there, where we are not even talking about health promotion or prevention yet.”* – EDB7, director, 35–39y, 
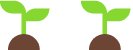

Some of the interventions were also noted for their calming effects before bedtime. In one kindergarten, the daily use of aromatherapy was reported by teachers and witnessed in the observations to support children in calming down and falling asleep (22).

However, some challenges were identified both by interview participants and through focused ethnographic observations. In line with the salutogenic approach, examining these in relation to their potential for health promotion is crucial to provide insights into how environmental, organizational, and individual factors may support or hinder the development of a SOC among teachers and children.

### 3.3 Challenges encountered throughout the implementation

The following section outlines the challenges identified in the data and describes how initial skepticism, structural questions, and emerging problems were addressed throughout the KC’s implementation. The main categories are summarized in Figure 2: Implementational Challenges.

**Figure 2:**
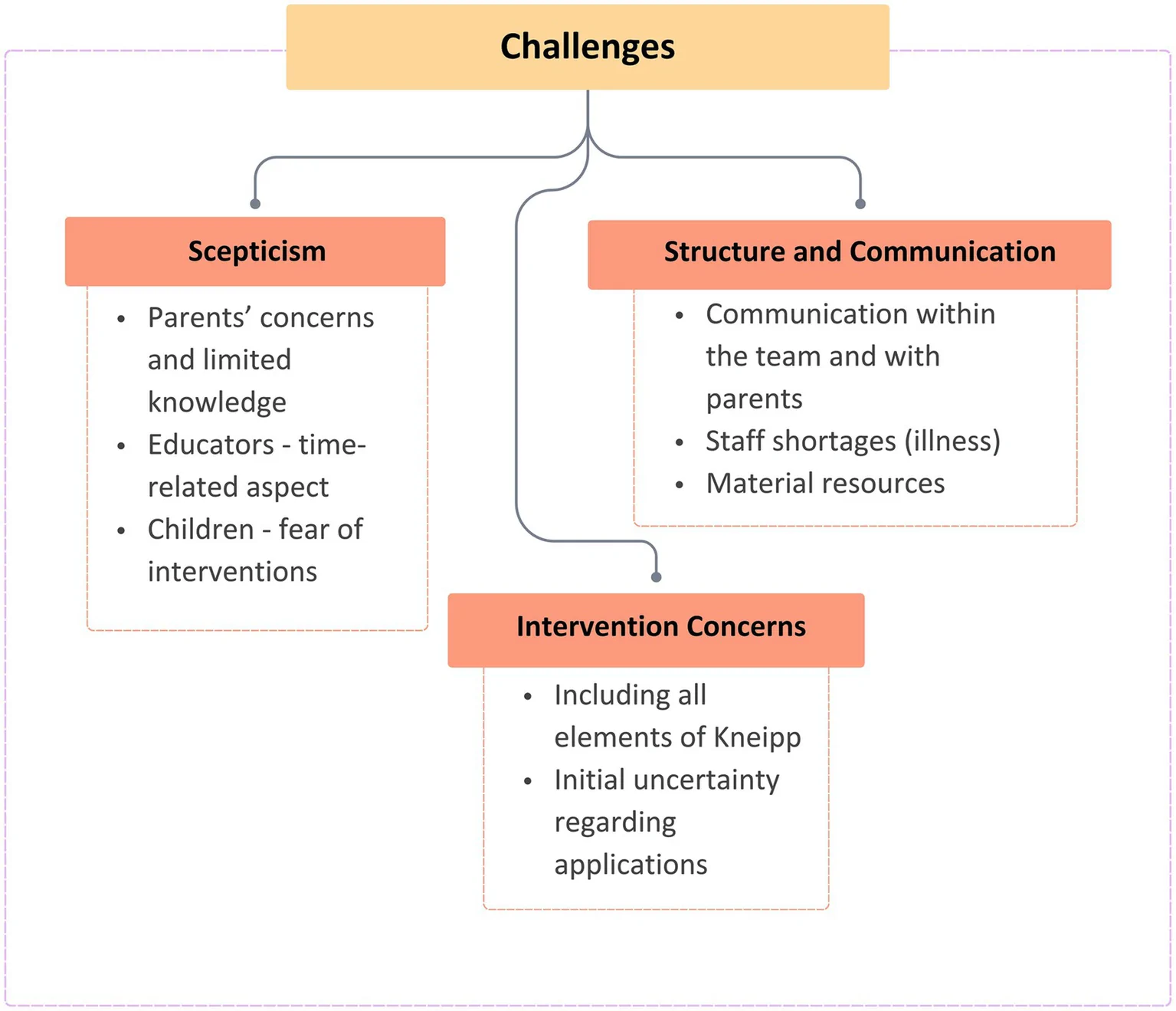
Implementational challenges – Key categories and subcategories drawn from data analysis.

#### 3.3.1 Initial skepticism

Some teachers noted initial reservations regarding the concept, often given lack of experience or concerns about potential time constraints. This was particularly evident in kindergarten facilities where teachers were not involved in the initial decision to participate in the study that required KC activity integration in the intervention facilities. After gaining experience through implementation of Kneipp practices and seeing the positive influences on children’s health, self-awareness and interpersonal relationships, this challenge was then dispelled.

*“At first, it was kind of forced by the previous director. She was always enthusiastic about the concept and was already familiar with it […] But like I said, at first, it felt more forced […] And now, we actually enjoy doing it.”* – SBA3, director, 35–39y, 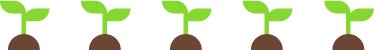

Additionally, some children initially resisted participation, particularly in cold-water applications. In observations and interviews, it was highlighted that children’s interest and motivation grew after observing peers enjoying activities and by gaining more experience with the interventions, by getting used to the cold water after participating several times.

*“The only thing (that children asked) was why we had to use cold water. They actually thought we would be using lukewarm water or something like that. And then it was like: ‘No, we’re doing it with cold water.’ At first, they were like, ‘Ah, it’s so cold!’ — but they got used to it very quickly. After the first few applications, after the first few weeks, they didn’t even check the temperature anymore.”* - ECB9, teacher, 25–29y, 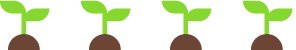

*“There were few children who didn’t want to go into the water – among them a boy who stood next to it the whole time and watched curiously. I (MG) asked him if he wanted to go into the pool– he shook his head “no”. But in the end, when almost all the children were out, he looked at me, pointed at the pool, and said: “Me too!” Then he went in with one of the teachers and did not want to get out of the water anymore.” -* PES, Summer observation- SBA

Teachers and directors underscored and highlighted how as regular KC practice increased, so did familiarity, confidence and expertise among the children. They described how children became ‘experts’ in Kneipp over time. In one kindergarten, for example, it was reported that they proudly showed and introduced some parents and children from other kindergartens to the concept at a summer festivity.

Other motivational factors overcoming hesitancy were children’s own recognition of the positive effects of Kneipp, the encouragement of trusted teachers, and the excitement of independence, and exploring new fun experiences alone or in a group. These examples illustrate how the KC supported salutogenesis by fostering SOC, helping children make sense of activities (comprehensibility), feel capable of engaging with them (manageability), and perceive them as valuable (meaningfulness) through a combination of internal resources and supportive external factors.

#### 3.3.2 Structural challenges and communication

Challenges regarding facility structures such as staff shortages due to illness, lack of time, and equipment were noted.

*“In winter, so many colleges were sick, and many had colds and coughs. So, we said we will focus on dry brushing and herbs for now and try to introduce that and we will do water again when it is a bit warmer.”* – SBA6, teacher, 35–39y, 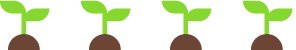

The importance of good communication and teamwork was often noted by interviewees when it came to organizing activities. In one of the facilities, staff who were practicing KC activities felt overwhelmed by the initial lack of involvement of other teachers and having responsibility for so many children during KC activities.

*“I was responsible for [implementing KC in] the whole house at first. But you really notice that it is too much. You simply need support. You need someone else in such a big facility who can also take the lead.”* – WCB4, teacher, 60–4y, 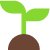

Clear communication and the active involving of new teachers—by inviting them to observe and experience the simplicity of the interventions—helped overcome this challenge and facilitated the continued implementation of the concept.

#### 3.3.3 Problems with parents

Although parents in most facilities were reported to have a positive attitude toward the introduction of Kneipp, notable challenges emerged in kindergartens particularly in lower socio-economic areas. In one kindergarten, an informational evening held prior to the intervention was poorly attended, and lack of participation led to misconceptions and concerns among parents about some interventions, for example barefoot snow treading.

*“And yes, a bit of work with the parents was necessary. I also had another conversation with one mom. She was very dissatisfied because she said a neighbor [living next to the kindergarten] had told her: ‘the children are running around barefoot [in the snow] in the garden for hours.’”* – EDB1, teacher, 50–54y, 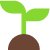

Active parental involvement was mentioned as a crucial factor for the successful integration of the KC into daily practice, next to the acceptance and motivation among teachers and children who practice regularly. These factors: motivation, consistency, and parental engagement—were found to be essential for the long-term sustainability of the KC.

## 4 Discussion

This study investigated salutogenesis and prevention through the prism of the KC. This analysis found that the KC can be meaningfully integrated into kindergarten settings by strengthening key resources such as mindfulness, social competence, and resilience. These resources are closely linked to the development of a SOC and underline the relevance of the KC as an early health-promoting intervention in educational contexts. As reducing health inequities is a key objective of primary health care, providing children from diverse socioeconomic backgrounds with access to such resources represents an important opportunity early in life to support this vulnerable population with practices that potentially promote long-term health over the course of a lifetime.

At the same time, the challenges of implementing such programs in childcare facilities cannot be overlooked and must be addressed to ensure their effective and lasting use for promoting children’s health. Our findings further indicate that the successful implementation of the KC depends on several key factors. Consistent with previous research, regular communication with parents and ongoing staff training were, for example, identified as key strategies (33). Literature on early childhood interventions recommend involving the entire staff and teachers actively participating in activities alongside children, to enhance motivation for behavioral change in the context of salutogenesis (34).

There is research on health promoting programs being implemented in childcare settings, where the importance of including parents in this process was highlighted (35). The absence of parents’ direct input on the KC is an acknowledged key limitation to our study. However, in another currently-conducted kindergarten Kneipp-related research project in Bavaria, Germany, parent perspectives are being explored and may provide an important viewpoint to KC activities (23). Further, Kneipp training and equipment provision afforded by the project may have led teachers to a positive bias in their reporting of Kneipp activities despite assurances to speak freely about their experiences. A long-term follow-up period would be helpful to see how and if Kneipp activities continue to be practiced.

This study’s mixed-methods approach constitutes a major strength, given that SOC research in salutogenesis profits from combined quantitative and qualitative methodologies, as highlighted in the Handbook of Salutogenesis (36). Moreover, the triangulation between data from interviews and focused ethnographies allowed us to closely follow the process of integrating such a holistic concept into already established childcare settings.

Studies with long-term follow-ups have already found out that children with psychological problems at an early age have poorer SOC-score in their adolescent life (37). Research also shows that SOC levels can be positively influenced by different interventions for preschoolers (38, 39). In this sense conducting a future study on the impact of the Kneipp intervention in a long-term follow-up would be beneficial to better examine if the concept affects sustainably children’s physical and mental health, as well as health-related/salutogenic behaviors later in their life. It would be also valuable to examine whether this concept can be successfully transferred to other childcare settings, such as schools (40).

Overall, this study highlights the KC as a promising salutogenic approach in early childhood education, demonstrating how health-promoting resources can be fostered within everyday pedagogical practice. The physical and mental benefits, development of self-regulation, or overcoming shared challenges with peers, were observed after practicing simple KC interventions such as cold-water applications or dry brushing. This is emblematic of SOC and helps build resilience in preschoolers by increasing practices that can be sustainably embedded in early educational settings. By linking theory with lived institutional experience, the findings contribute to a deeper understanding of how such a holistic concept can be integrated in real life to improve health awareness supporting mental and physical health of children that are important to primary health.

## Data Availability

The raw data supporting the conclusions of this article will be made available by the authors, without undue reservation.
